# Bone marrow fibrosis grade is an independent risk factor for overall survival in patients with primary myelofibrosis

**DOI:** 10.1038/bcj.2016.116

**Published:** 2016-12-09

**Authors:** B Li, P Zhang, G Feng, Z Xu, T Qin, Y Zhang, Z Sha, D Dong, H Zhang, L Fang, L Pan, N Hu, S Qu, W Cai, G Huang, Z Xiao

**Affiliations:** 1 MDS and MPN Centre, Institute of Hematology and Blood Diseases Hospital, Chinese Academy of Medical Sciences and Peking Union Medical College, Tianjin, China; 2 State Key Laboratory of Experimental Hematology, Institute of Hematology and Blood Diseases Hospital, Chinese Academy of Medical Sciences and Peking Union Medical College, Tianjin, China; 3 Department of Pathology, Institute of Hematology and Blood Diseases Hospital, Chinese Academy of Medical Sciences and Peking Union Medical College, Tianjin, China; 4 Divisions of Experimental Hematology and Cancer Biology, Cincinnati Children's Hospital Medical Center, Cincinnati, OH, USA

Histopathological findings have a key role in diagnosis of primary myelofibrosis (PMF). According to the revised 2016 World Health Organization (WHO) classification of PMF,^1^ bone marrow (BM) fibrosis represents a major diagnostic criteria together with abnormal megakaryocyte morphology. The European consensus^2^has been applied to evaluate the BM fibrosis grade in the revised 2016 WHO classification. According to the European consensus, fibrosis is graded in four levels, from grade 0 to grade 3.Moreover, PMF is further divided into prePMF (MF-0 or MF-1) and overt PMF (MF-2 or MF-3) according to fibrosis grade.^1^ Although BM fibrosis is a major criteria for PMF, the fibrosis grade is not incorporated in conventional prognostic scoring systems. Recently, it is emphasized that an accurate evaluation of BM fibrosis grade has been proven to be a key point to predict prognosis in PMF.^3, 4, 5^ In this study, we re-evaluated the diagnostic biopsies of 330 patients with PMF and analyzed the prognostic impact of addition of fibrosis grade in the traditional prognostic scoring system.

In 330 patients, 235 (71.2%) were at diagnosis and 95 (28.8%) at referral. The median time between the original diagnosis and the referral was 36 (5–132) months. No patient had received hematopoietic stem cell transplantation. All patients had a high-quality biopsies collected at diagnosis or referral and gave informed consent compliant with the Declaration of Helsinki. All cases were blind re-reviewed by two experienced pathologists and reclassified based on the revised 2016 WHO classification. Dynamic International Prognostic Scoring System (DIPSS)^6^ were calculated as described. One hundred and ninety-five patients had evaluable cytogenetic results. According to DIPSS-plus,^7^ karyotypes were classified as the favorable and the unfavorable. *JAK2*, *CALR* and *MPL* mutations were tested at diagnosis as described.^8^ Follow-up data were available for 301 patients, and the median follow-up was 39 (1–255) months. Correlations between sample groups and clinical and laboratory data were calculated with the χ^2^ test for qualitative variables with discrete categories and Mann–Whitney *U*-test or Kruskal–Wallis analysis of variance for continuous variables. Survival distribution was estimated by the Kaplan–Meier method and was compared between subgroups using the log-rank test. The Cox proportional hazards regression model was used to assess the correlation between variables and survival. Two-tailed *P*-values⩽0.05 were considered significant.

In 330 patients, 75 (22.7%) were categorized as DIPPS low-risk group, 154 (46.7%) intermediate-1-risk group, 93 (28.2%) intermediate-2-risk group and 8 (2.4%) high-risk group. *JAK2* mutations were detected in 162 subjects (49.1%), *CALR* mutations in 65 (19.7%), *MPL* mutations in 8 (2.4%) and triple-negative (no detectable mutation in *JAK2*, *CALR* or *MPL*) in 95 (28.8%). According to the European consensus, 14 (4.2%) had MF-0, 93 (28.2%) MF-1, 165 (50%) MF-2 and 58 (17.6%) MF-3. Compared with the patients with MF-0 or MF-1, patients with MF-2 or MF-3 were older (*P*=0.014), had more frequent hemoglobin concentrations <100 g/l (*P*<0.001), less frequent WBC levels >25 × 10^9^/l (*P*=0.028), more frequent platelet levels <100 × 10^9^/l (*P*=0.017), higher DIPSS scores (*P*<0.001) and more frequent unfavorable karyotype according to DIPSS-plus (*P*=0.017). However, the fibrosis grade was not associated with the size of splenomegaly and driver mutations. There were more patients with MF-2 or MF-3 who died at last follow-up than patients with MF-0 or MF-1 (31.2% versus 13.1% *P*<0.001). Supplementary Tables S1 and S2 list baseline clinical and laboratory variables of the 330 study subjects categorized by BM fibrosis grade.

In univariate analysis, patients with higher fibrosis grade had shorter overall survival (OS) (*P*=0.013, Supplementary Figure S1A). Patients with overt fibrosis (MF-2 or MF-3) had significantly shorter OS compared with subjects with prefibrosis (MF-0 or MF-1) (*P*=0.001, Supplementary Figure S1B). Moreover, DIPSS variables (*P*<0.0001), no palpable splenomegaly (*P*=0.004), thrombocytopenia (*P*<0.001) and CALR-type-2 or triple-negative mutation (*P*<0.001) were associated with reduced OS.

In the lower-risk DIPSS group (low- and intermediate-1-risk group), MF-2 or MF-3 identified patients with shorter OS compared with MF-0 or MF-1 (*P*=0.014, Supplementary Figure S1C) while for patients in the higher-risk group (intermediate-2- and high-risk group), fibrosis grade had no impact on OS (Supplementary Figure S1D).

In multivariable Cox proportional hazard regression analysis (Table 1), MF-2 or MF-3 remained significant for OS (hazard ratio (HR): 2.51, 95% confidence interval (CI): 1.37–4.59; *P*=0.003) together with DIPSS variables (HR: 2.40, 95% CI: 1.64–3.51; *P*<0.001), no palpable splenomegaly (HR: 1,72, 95% CI: 1.03–2.86; *P*=0.036), thrombocytopenia (HR: 2.65, 95% CI: 1.62–4.34; *P*<0.001) and CALR-type-2 or triple-negative mutation (HR: 1.82, 95% CI: 1.10–3.02; *P*=0.02).

Based on these data, we developed a new prognostic model using the HRs defined in the Cox regression. We assigned each factor a weight: (1) 2 for DIPSS high risk; (2) 1 for DIPSS intermediate-2 risk and platelets <100 × 10^9^/l; (3) 0.5 for no splenomegaly and CALR-type-2 or triple-negative mutation. Patients were categorized into four risk cohorts: (1) low (0–1); (2) intermediate-1 (1.5 and 2); intermediate-2 (2.5 and 3); and high (⩾3.5). One hundred and thirty-one subjects (39.7%) were categorized into low-risk cohort, 100 (30.3%) intermediate-1-risk cohort, 68 (20.6%) intermediate-2-risk cohort and 31 (9.4%) high-risk cohort. The median OS for the four risk categories was not reached, 240, 72 and 18 months, respectively, and the difference was highly significant (*P*<0.001; Figure 1). Compared with estimated HRs for survival in the low-risk cohort, HRs were 2.94 (95% CI, 1.45–5.99; *P*=0.003) for the intermediate-1-risk cohort, 6.18 (95% CI, 3.05–12.52; *P*<0.001) for the intermediate-2-risk cohort and 22.70 (95% CI, 10.81–47.67; *P*<0.001) for the high-risk cohort.

Histopathological findings are major criteria for PMF together with polycythemia vera and essential thrombocythemia (ET) according to the revised 2016 WHO classification.^1^ Hematopoietic cellularity, granulocytic, erythrocytic and megakaryocytic proliferation, abnormal arrangement, location and morphology of megakaryocyte and reticulin and/or collagen fibrosis are key points concerning the distinction between polycythemia vera, ET, prePMF and overt PMF.^9, 10^ Reproducibility and clinical usefulness of the WHO classification to differential diagnosis for Ph− myeloproliferative dysplasia persisted to be a controversial issue in recent years. Although some studies offered some criticisms of WHO morphological classification,^11, 12^ a number of clinico-pathological studies by independent working groups demonstrated that definite diagnosis could be made by strictly regarding histopathological features according to the WHO criteria.^13, 14^ Compared with ET and overt PMF, prePMF has unique clinical and laboratory features and outcome.^15^ Therefore, discriminating prePMF from ET and overt PMF is necessary and accurate evaluation of BM fibrosis grade is a key issue to diagnosis and prognostic evaluation for prePMF.

Our study indicated that higher BM fibrosis grade was associated with some poor prognostic characteristics, including older age, anemia, thrombocytopenia, unfavorable karyotype and a higher DIPSS risk category, but fibrosis grade was not associated with driver mutations. Multivariable analyses confirmed that fibrosis grade was independent of DIPSS score for PMF patients, especially in the lower-risk group.Findings from this study agreed with previous studies.^3, 4, 5^ This study indicated that there were obvious differences in clinical characteristics and prognosis between prePMF (MF-0 or MF-1) and overt PMF fibrosis (MF-2 or MF-3) as currently defined by WHO. Therefore, adding fibrosis grade into the traditional prognostic scoring system is necessary to accurate evaluation of prognosis.

In conclusion, we confirmed the independent prognostic impact of fibrosis grade in PMF and the important clinical meaning of the revised 2016 WHO classification for PMF. The main limitation of this study is the lack of validation in an independent cohort of the proposed score; ideally this score system should be validated in another data set in the future.

## Figures and Tables

**Figure 1:**
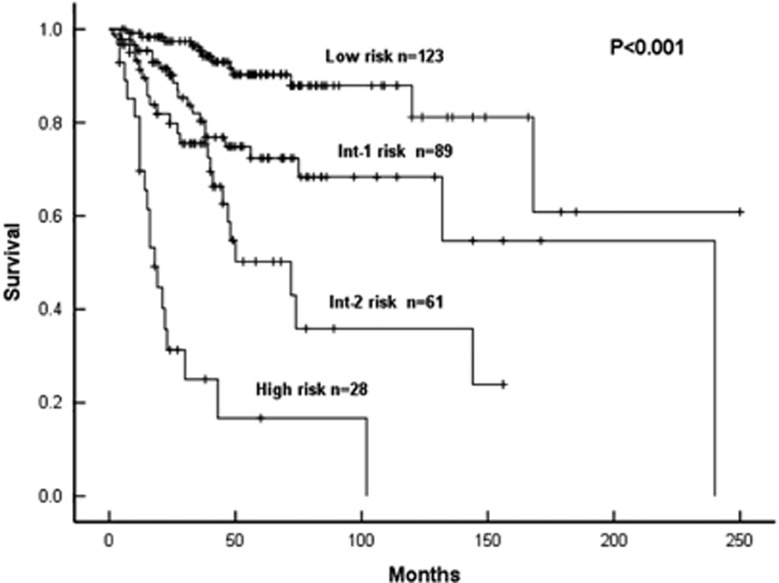
Kaplan–Meier curves of OS in 301 patients according to bone marrow fibrosis grade together with DIPSS variables, no palpable splenomegaly, thrombocytopenia and CALR-type-2 or triple-negative mutations.

**Table 1:** Multivariable Cox proportional hazard regression analysis in 301 patients with primary myelofibrosis

*Variable*	*HR*	*95% CI*	P	*Score*
DIPSS intermediate-2-risk group	2.31	1.43–3.73	0.001	1
DIPSS high-risk group	6.17	2.50–15.23	<0001	2
Platelets <100 × 10^9^/l	2.65	1.62–4.34	<0.001	1
No palpable splenomegaly	1.72	1.03–2.86	0.036	0.5
*CALR*-type-2 or triple-negative mutation	1.82	1.10–3.02	0.02	0.5
Fibrosis grade 2 or 3	2.51	1.37–4.59	0.003	1

Abbreviations: CI, confidence interval; DIPSS, Dynamic International Prognostic Scoring System; HR, hazard ratio.
