# Supplementary material for: Bone marrow fibrosis grade is an independent risk factor for overall survival in patients with primary myelofibrosis
Source: Blood Cancer J. 2016 Dec 9;6(12):e505–. doi: 10.1038/bcj.2016.116 (PMC5223151; doi:10.1038/bcj.2016.116)

**Supplementary Table 1.Clinical and laboratory features of 330 patients classified as bone marrow fibrosis grade.**

|  | **Bone marrow fibrosis** | | | |  |
| --- | --- | --- | --- | --- | --- |
| **Variable** | **Grade 0 (n=14)** | **Grade 1 (n=93)** | **Grade 2(n=165)** | **Grade 3(n=58)** | ***P*** |
| Age (y) median (range) | 48 (21-65) | 52 (15-81) | 58 (21-81) | 56 (26-89) | 0.035 |
| Males n (%) | 5 (35.7) | 50 (53.8) | 85 (51.5) | 26 (44.8) | 0.488 |
| HGB (g/L) median (range) | 125 (52-176) | 116 (30-182) | 90 (27-195) | 87 (35-148) | <0.001 |
| HGB<100g/L; n(%) | 4 (28.6) | 29 (31.2) | 98 (59.4) | 39 (67.2) | <0.001 |
| WBC (x10^9^/L) median (range) | 13.5 (2.2-39) | 13.3 (0.9-41.5) | 7.7 (0.6-46.2) | 8.4 (0.5-85.8) | 0.006 |
| WBC>25x109/L; n (%) | 2 (14.3) | 19 (20.4) | 20 (12.1) | 4 (6.9) | 0.101 |
| Platelets (x10^9^/L)median (range) | 287 (9-1246) | 296 (12-1373) | 163 (6-1129) | 134 (4-1083) | 0.005 |
| Platelets<100x109/L;n (%) | 4 (28.6) | 23 (24.7) | 66 (40) | 20 (34.5) | 0.089 |
| Peripheral blasts≥1%; n (%) | 3 (21.4) | 21 (22.6) | 52 (31.5) | 18 (31) | 0.401 |
| Constitutional symptoms; n (%) | 2 (14.3) | 18 (19.4) | 30 (18.2) | 10 (17.2) | 0.965 |
| Splenomagly from LCM (cm) median (range) | 5 (0-15.8) | 6 (0-28) | 5 (0-35) | 8 (0-30) | 0.059 |
| Splenomagly>5cm LCM; n (%) | 7 (50) | 52 (55.9) | 85 (51.5) | 41 (70.7) | 0.085 |
| Unfavorable Karyotype | 0 | 3 (6.4) | 19 (19) | 5 (14.3) | 0.035 |
| Driver mutations; n (%) |  |  |  |  | 0.518 |
| *JAK2V617F* | 7 (50) | 53 (57) | 72 (43.6) | 30 (51.7) |  |
| *MPLW515* | 0 | 1 (1.1) | 6 (3.6) | 1 (1.7) |  |
| *CALR* | 3 (21.4) | 14 (15.1) | 34 (20.6) | 14 (24.1) |  |
| Triple-negative | 4 (28.6) | 25 (26.9) | 53 (32.1) | 13 (22.4) |  |
| DIPSS risk group; n (%) |  |  |  |  | 0.009 |
| Low | 4 (28.6) | 32 (34.4) | 31 (18.8) | 8 (13.8) |  |
| Intermediate-1 | 9 (64.3) | 39 (41.9) | 76 (46.1) | 30 (51.7) |  |
| Intermediate-2 | 1 (7.1) | 21 (22.6) | 51 (30.9) | 20 (34.5) |  |
| High | 0 | 1 (1.1) | 7 (4.2) | 0 |  |

**Abbreviation:** HGB, hemoglobin; WBC white blood cell; LCM, left costal margin; DIPSS, Dynamic International Prognostic Scoring System.

**Supplementary Table 2.Clinical and laboratory features of 330patients classified as bone marrow fibrosis grade 0 or 1 and 2 or 3.**

|  | **Bone marrow fibrosis** | |  |
| --- | --- | --- | --- |
| **Variable** | **Grade 0-1 (n=107)** | **Grade 2-3 (n=223)** | ***P*** |
| Age (y) median (range) | 51 (15-81) | 57 (21-89) | 0.014 |
| Males n (%) | 55 (51.4) | 111 (49.8) | 0.782 |
| HGB (g/L) median (range) | 117 (30-182) | 89 (27-195) | <0.001 |
| HGB<100g/L; n(%) | 33 (30.8) | 137 (61.4) | <0.001 |
| WBC (x10^9^/L) median (range) | 13.3 (0.88-41.5) | 8 (0.5-85.78) | <0.001 |
| WBC>25x109/L; n (%) | 21 (19.6) | 24 (10.8) | 0.028 |
| Platelets (x10^9^/L)median (range) | 292 (9-1373) | 152 (4-1129) | <0.001 |
| Platelets<100x109/L;n (%) | 27 (25.2) | 86 (38.6) | 0.017 |
| Peripheral blasts≥1%; n (%) | 24 (22.4) | 70 (31.4) | 0.091 |
| Constitutional symptoms; n (%) | 20 (18.7) | 40 (17.9) | 0.868 |
| Splenomagly from LCM (cm) median (range) | 6 (0-28) | 6 (0-35) | 0.995 |
| Splenomagly>5cm LCM; n (%) | 59 (55.1) | 126 (56.5) | 0.815 |
| Unfavorable Karyotype | 3 (5) | 24 (17.8) | 0.017 |
| Driver mutations; n (%) |  |  | 0.202 |
| *JAK2V617F* | 60 (56.1) | 102 (45.7) | ; |
| *MPLW515* | 1 (0.9) | 7 (3.1) |  |
| *CALR* | 17 (15.9) | 48 (21.5) |  |
| Triple-negative | 29 (27.1) | 66 (29.6) |  |
| DIPSS risk group; n (%) |  |  | <0.001 |
| Low | 36 (33.6) | 39 (17.5) |  |
| Intermediate -1 | 48 (44.9) | 106 (47.5) |  |
| Intermediate -2 | 22 (20.6) | 71 (31.8) |  |
| High | 1 (0.9) | 7 (7.1) |  |

**Abbreviation:** HGB, hemoglobin; WBC white blood cell; LCM, left costal margin; DIPSS, Dynamic International Prognostic Scoring System.

**Supplementary Figure 1.Overall survival of 301 patients with PMF classified by bone marrow fibrosis grade.(A). MF-0 versus MF-1 versus MF-2 versus MF-3; (B).MF-0 or MF-1 versus MF-2 or MF-3 in all patients; (C).MF-0 or MF-1 versus MF-2 or MF-3 in the lower-risk DIPSS groups; (D). MF-0 or MF-1 versus MF-2 or MF-3 in the higher-risk DIPSS groups**

**A**


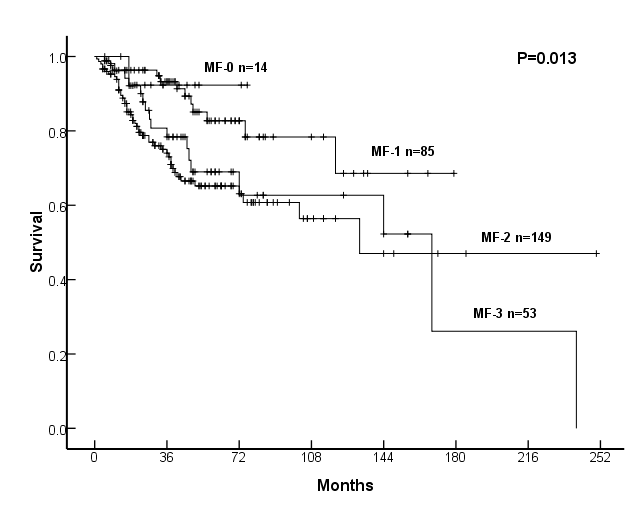


**B**


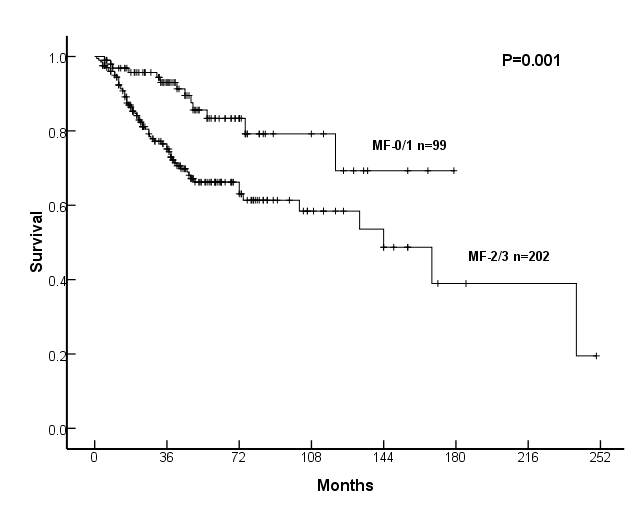


**C**


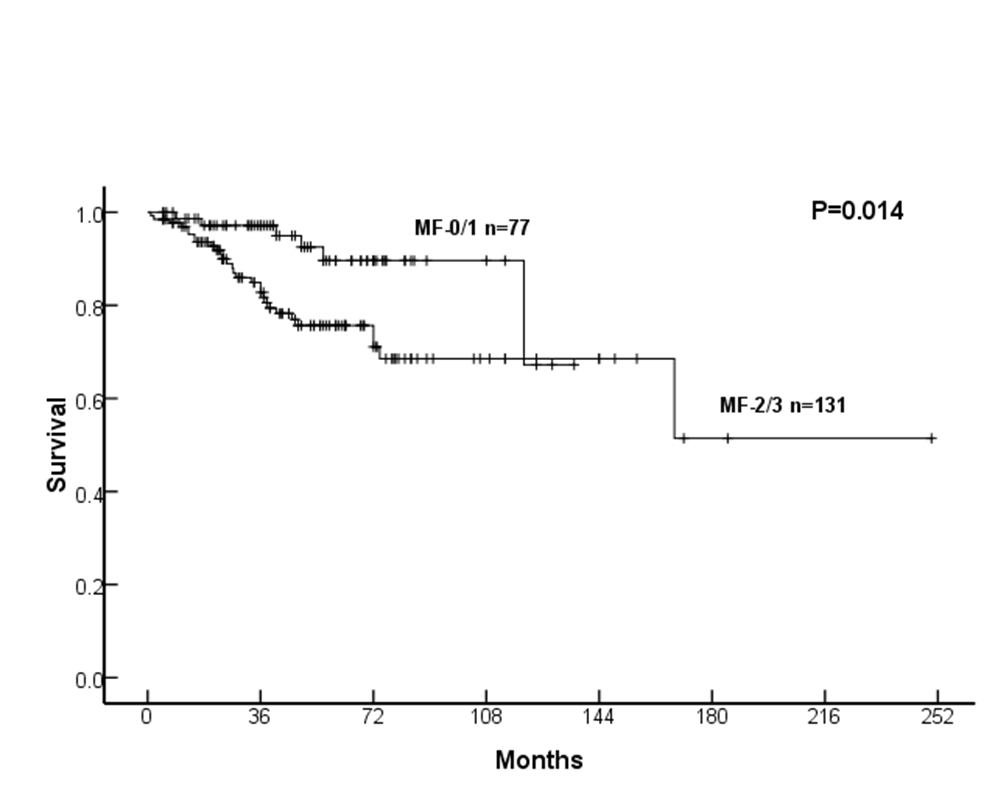


**D**


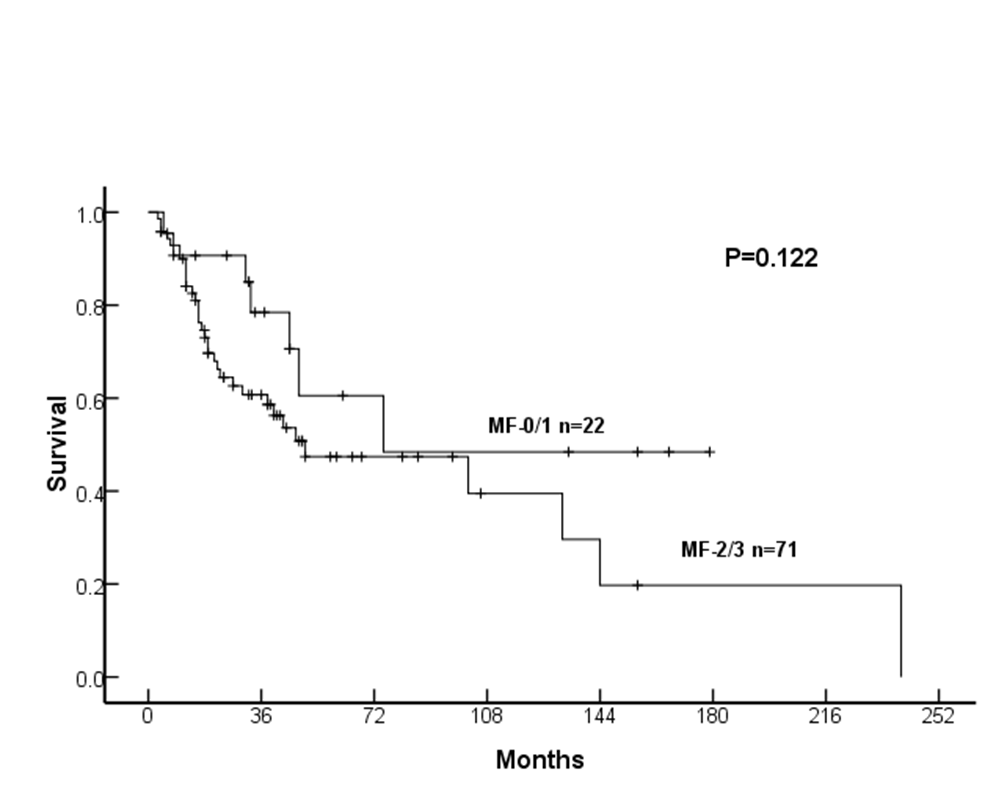

Supplement: Supplementary Tables and Figures [file bcj2016116x1.doc]
